# Facilitating Word Retrieval in Aphasia: Which Type of Cues for Which Aphasic Speakers?

**DOI:** 10.3389/fnhum.2021.747391

**Published:** 2021-11-26

**Authors:** Grégoire Python, Pauline Pellet Cheneval, Caroline Bonnans, Marina Laganaro

**Affiliations:** ^1^Faculty of Psychology and Educational Sciences, University of Geneva, Geneva, Switzerland; ^2^Department of Clinical Neurosciences, Neurorehabilitation Unit, University Hospital CHUV, Lausanne, Switzerland; ^3^Neurorehabilitation Unit, Lavigny Institution, Lavigny, Switzerland

**Keywords:** anomia, picture naming, semantic priming, phonological cueing, facilitation

## Abstract

**Background:** Even if both phonological and semantic cues can facilitate word retrieval in aphasia, it remains unclear if their respective effectiveness varies according to the underlying anomic profile.

**Aim:** The aim of the present facilitation study is to compare the effect of phonological and semantic cues on picture naming accuracy and speed in different types of anomia.

**Methods:** In the present within-subject design study, 15 aphasic persons following brain damage underwent picture naming paradigms with semantic cues (categorically- or associatively related) and phonological cues (initial phoneme presented auditorily, visually or both).

**Results:** At the group level, semantic cueing was as effective as phonological cueing to significantly speed up picture naming. However, while phonological cues were effective regardless of the anomic profile, semantic cueing effects varied depending on the type of anomia. Participants with mixed anomia showed facilitation after both semantic categorical and associative cues, but individuals with lexical-phonological anomia only after categorical cues. Crucially, semantic cues were ineffective for participants with lexical-semantic anomia. These disparities were confirmed by categorical semantic facilitation decreasing when semantic/omission errors prevailed in the anomic profile, but increasing alongside phonological errors.

**Conclusion:** The effectiveness of phonological *vs* semantic cues seems related to the underlying anomic profile: phonological cues benefit any type of anomia, but semantic cues only lexical-phonological or mixed anomia.

## Introduction

For several decades, different kinds of semantic or phonological cues have been used in aphasia research to investigate their (often facilitative) effects on impaired word production. First studies comparing both facilitation techniques within the same aphasic individuals suggested an advantage of semantic cueing over phonological cueing (Howard et al., 1985). However, subsequent facilitation studies either found comparable effectiveness of these two types of cues (Stimley and Noll, 1991) or claimed that phonological cueing was overall more effective than semantic cueing (Saito and Takeda, 2001; Meteyard and Bose, 2018). A recent computational model simulating naming tasks also concluded that phonological cues could potentially induce greater facilitation than semantic cues (Stille et al., 2020). In fact, the underlying impairment may modulate the sensitivity to semantic *vs* phonological cues, as it has been reported that phonological cueing was particularly effective for persons with Broca and conduction aphasia, while semantic cueing led to better responses in persons with anomic aphasia (Li and Williams, 1990). In the aforementioned facilitation studies, analyses were carried out only on accuracy, without investigating naming latencies. Here, the aim is to identify which types of phonological or semantic cues are most facilitative for immediate word retrieval in aphasic speakers according to their underlying anomic profile, both in terms of errors and naming latencies.

Phonological cueing often consists in providing the first phoneme(s) or the rhyme of the target-word, whereas semantic cueing refers to a wide range of situations, such as giving the superordinate word (e.g., cue “vegetable” to name the asparagus), an associative verb (e.g., cue “you ring it” to name the bell) or/and definitions or sentences to complete (e.g., cue “a farm animal that gives milk/the farmer went to the barn to milk the…” to name the cow) (e.g., Li and Williams, 1990). Semantic cueing combines different kinds of semantic information, likely associated with different stages of word retrieval. For instance, associative relationships might be exclusively related to features at the semantic/conceptual level, whereas categorical relationships also to lexical co-activated entries/competitors. That is why Meteyard and Bose (2018) proposed that future research should compare different types of semantic cues to better understand semantic priming in word production.

In psycholinguistic studies exploring semantic and phonological facilitation, the picture-word interference paradigm has been widely used in healthy controls (e.g., Schriefers et al., 1990; Peterson and Savoy, 1998; Jescheniak and Schriefers, 2001; Mahon et al., 2007) and to a more limited extent in aphasic speakers (e.g., group studies of Best et al., 2002; Python et al., 2018b). In this paradigm, the time interval between the presentation of the priming word and the target-picture, called Stimulus Onset Asynchrony (SOA), is a crucial variable (Bürki et al., 2020). Semantic facilitation is typically induced only by long negative SOAs, i.e., words presented at least −400 ms before the picture (e.g., Zhang et al., 2016; Python et al., 2018a) whereas semantic interference is often observed at shorter SOAs (see Mahon et al., 2007, for a review). This polarity reversal indicates that different processes can come into play in semantic cueing paradigms and may therefore lead to inconsistent results with aphasic participants depending on the type of semantic cue and/or the underlying impairment. In phonological cueing, the default pattern seems less prone to reversal as facilitation can occur with phonologically related words presented from −300 ms before to +150 ms after the picture (e.g., Starreveld, 2000).

In sum, both phonological and semantic cueing techniques have proved beneficial for aphasic speakers, but straightforward links between the underlying anomic profile and the effects of the provided cue (semantic vs. phonological) have not been established. Actually, the relationship between the underlying impairment and the processes targeted by anomia treatments administered over several weeks also remains ambiguous (Wambaugh et al., 2001; Lorenz and Ziegler, 2009; Best et al., 2013; van Hees et al., 2013). Although facilitation studies and treatment studies cannot be directly compared, it has been shown that response to phonological facilitation significantly correlated with phonological treatment outcomes (Hickin et al., 2002).

The aims of the present facilitation study are:

(1) to compare the effectiveness of phonological cueing and semantic cueing on naming accuracy and production latencies in a group of aphasic speakers;
(2) to determine whether the effectiveness of different types of cues (two types of semantic cues and three types of phonological cues) is related to the underlying anomic profile (lexical-semantic, lexical-phonological or mixed anomia).

## Materials and Methods

### Participants

Fifteen aphasic persons aged 21–75 (mean 49 years) participated in this study (nine males) (Table 1). They were eligible if they were French-speaking, right-handed and were attending speech and language therapy sessions due to aphasia following brain damage. The co-occurrence of neurodegenerative or psychiatric diseases was an exclusion criterion, as well as severe associated motor speech disorders. Participants were recruited in 2015–2016 from Neurorehabilitation Units either in Lausanne or in Lavigny, Switzerland. Aphasia was diagnosed by experienced speech and language pathologists: eleven persons were classified as fluent speakers, three as non-fluent (P6, P12, and P14) and one was not classifiable (P10). Minimal information about their language profile prior to this study is reported here, because no specific battery was consistently administered to all participants and heterogeneous diagnostic tools were used by the clinicians. Nevertheless, all participants suffered from mild to moderate anomia as determined by the French shortened version of the Boston Naming Test (Thuillard Colombo and Assal, 1992) (see scores in Table 1). Written informed consent was given by all participants and approved by the local ethical research committee (CER-VD, in accordance with the Declaration of Helsinki). Brain damage was due to stroke in 13 of the participants, cyst resection in one case and traumatic brain injury in one case (Table 1).

**TABLE 1:** Demographic, lesion and behavioral data of the 15 aphasic individuals.

**Participant**	**Age**	**Gender**	**Etiology and lesion location**	**TPO**	**BNT**
P1	59	F	Left posterior stroke	3	97%
P2	39	M	Left frontal stroke	2	74%
P3	21	M	Left sylvian stroke	8	88%
P4	51	M	Left sylvian stroke	2	97%
P5	48	M	Left multi-focal stroke	12	71%
P6	37	F	Left sylvian stroke	27	91%
P7	52	M	Left multi-focal stroke	19	85%
P8	52	M	Left sylvian stroke	37	85%
P9	60	M	Left sylvian stroke	25	88%
P10	29	F	Left mesial temporal cyst resection	27	91%
P11	65	F	Left sylvian stroke	8	95%
P12	26	F	Fronto-temporal due to TBI	62	68%
P13	75	F	Left frontal stroke	3	75%
P14	62	M	Left sylvian stroke	4	97%
P15	57	M	Left multi-focal stroke	1	88%

*TBI, Traumatic Brain Injury; TPO, Time Post Onset (in months); BNT, picture naming accuracy at the shortened French version of the Boston Naming Test (*n* = 34).*

### Materials and Procedure

All participants underwent two paradigms: a phonological cueing paradigm before a semantic cueing paradigm (see below). This fixed order was meant to avoid unwanted effects of the semantic paradigm over the phonological paradigm: as semantic effects could be temporally persistent (Howard et al., 2006; Oppenheim et al., 2010), the paradigm with shorter-lasting effects (i.e., the phonological cueing paradigm) was administered first. For both paradigms, the pictures were chosen in two databases normed in French (Alario and Ferrand, 1999; Bonin et al., 2003). The target-words to produce across the two paradigms were comparable in terms of lexical frequency, word length (number of phonemes/syllables) and phonological neighborhood density (all p*s* > 0.05; psycholinguistic properties retrieved from New et al., 2004).

#### Phonological Cueing Paradigm (see Supplementary Appendix I)

Thirty black and white line drawings (e.g., balloon) were associated with different phonological cues in three related conditions: videoclips depicting the initial phoneme (e.g.,/b/) presented auditorily (without lip movement), visually (without sound) or both. The neutral/unrelated condition was a videoclip with a static face and white noise. In total, 120 experimental trials were presented to each participant (30 pictures x 4 conditions) interspersed with 10 filler pictures preceded by incongruent cues. Pictures had a mean name agreement of 91.18% (SD = 10.97) and were not used in the semantic cueing paradigm. After a fixation cross (500 ms), the cue was played for 2000 ms and the picture to name was presented 100 ms after the end of the videoclip. The picture remained on screen for 2000 ms and the participants had 3000 ms to name it.

#### Semantic Cueing Paradigm (see Supplementary Appendix II)

Twenty-one black and white line drawings (e.g., spider) were associated with different semantic cues in two related conditions: auditory words either from the same category (e.g., ant) or frequent associates from a different semantic category (e.g., cobweb). Category-related cues were selected in Bueno and Megherbi (2009) and associatively related cues in Ferrand and Alario (1998). As a control condition, unrelated cues (e.g., jacket) were presented to the participants, that consisted in related cues re-paired to match unrelated targets. Pictures had a high name agreement in French (mean 91.93%, SD = 10.14). None of the word cues shared the initial or the final phoneme with the target picture to name. Each picture was preceded either by one or two word cues in a pseudo-random order, leading to 126 trials for each participant (21 pictures × 3 conditions × 2 number of cues). When two cues were played, they were separated by a 150 ms blank interval and were both unrelated, category-related or associatively related to the picture. Because the number of cues was not the focus or the current investigation, only the semantic condition (i.e., category- or associatively related) was retained as an experimental factor in subsequent analyses independently of the number of cues. After a fixation cross (250 ms), the cue(s) was/were played auditorily and the picture to name was finally presented 150 ms after the end of the cue(s). The picture remained on the display for 2000 ms and the participants had 3000 ms to give its label.

For both paradigms, aphasic persons were seated next to a speech and language therapist in front of a computer screen. E-Prime software^1^ was used to deliver the stimuli and record the responses of the participants up to three seconds after the presentation of the pictures. Every trial was manually launched by the experimenter. Before each paradigm, aphasic individuals were familiarized with all the pictures and their expected names. During this familiarization phase, the correct response was provided by the speech and language therapist in case of errors. No help was provided for word retrieval during the cueing paradigms. Participants were asked to name the pictures as quickly and accurately as possible. It lasted approximately 1 h to perform 246 naming trials in total with multiple breaks.

### Analyses

Utterances not corresponding to the expected single target name were considered as errors and classified as follows: omissions (i.e., no response within the time limit), circumlocutions (e.g., “to drive in town” for “car”), unrelated errors (e.g., “mouse” for “hammer”), formal errors (e.g., “carrot” for “parrot”), semantic errors (e.g., “sock” for “shoe”), or phonological errors (phoneme inversion, e.g., “dubstin” for “dustbin,” phoneme substitution, e.g., “rustbin” for “dustbin,” phoneme omission, e.g., “dusbin” for “dustbin” or phoneme addition, e.g., “drustbin” for “dustbin”). Participants were divided in three subgroups according to their profile of errors throughout the two paradigms: individuals producing a majority of phonemic errors were assigned to the lexical-phonological subgroup, individuals producing a majority of omissions/semantic errors to the lexical-semantic subgroup and individuals not exceeding 50% of errors in these categories to the mixed subgroup. Note that omission errors were included in the lexical-semantic profile as they are most likely due to pre-lexical impairments, either sharing a common source with semantic errors (Bormann et al., 2008) or corresponding to units’ activations unable to reach the lexical selection threshold (Dell et al., 2004). Recent computational modeling and lesion-symptom mapping data also speak in favor of a lexical-semantic origin of omission errors (Tochadse et al., 2018; Chen et al., 2019).

The effects of the cues on accuracy were calculated for each participant by subtracting the mean error rate of the control condition from the mean error rate of a given related condition. For correct trials only, naming latencies were defined manually with the software CheckVocal (Protopapas, 2007) and the reaction time data was log-transformed to approximate a normal distribution. The effects of the cues on naming latencies were calculated by subtracting the mean latencies of the control condition from the mean latencies of a given related condition, divided by the mean of the control condition. Therefore, negative proportions expressed facilitation induced by the cues whereas positive proportions interference.

Generalized mixed models (Jaeger, 2008) for errors, linear regression mixed-effects models (Baayen et al., 2008) and simple linear regressions for naming latencies were computed in R software (R Development Core Team, 2003). If models failed to converge, the Bound Optimization BY Quadratics Approximation was used instead of the default Nelder-Mead optimization algorithm (Powell, 2009). The alpha criterion was set to ≤0.05 in order to reject the null hypothesis and consider the results as significant.

## Results

### Accuracy

Among the 3690 observations, 412 were classified as errors (11.2%). Participants individually produced 1.2 to 27.2% errors. The mean error rate was 12.3% in the phonological cueing paradigm (range 0.8 to 25%) and 10.0% in the semantic cueing paradigm (range 0 to 29.4%). Errors were mainly phonemic (43%), omissions (20%), and semantic (15%). According to the subgroup assignment procedure (see above), five individuals showed a lexical-phonological profile (Table 2, column %PHO > 50%), five individuals a lexical-semantic profile (Table 2, column %SEM > 50%) and the five remaining individuals a mixed profile.

**TABLE 2:** Amount and type of errors throughout the two paradigms and proportion of phonemic, semantic/omission errors in both paradigms for the 15 participants and subgroup assignment.

**Participant**	**Raw number of errors**				**Proportion of errors**		**Error profile**
	**Phonemic**	**Semantic**	**Omission**	**Other**	**%PHO**	**%SEM**	
P1	46	1	4	2	86.8%	9.4%	L-phon
P2	6	1	1	2	60.0%	20.0%	L-phon
P3	4	6	4	1	26.7%	66.7%	L-sem
P4	7	0	0	0	100.0%	0%	L-phon
P5	7	5	1	7	35.0%	30.0%	Mixed
P6	0	1	14	6	0%	71.4%	L-sem
P7	5	2	0	3	50.0%	20.0%	Mixed
P8	13	8	41	5	19.4%	73.1%	L-sem
P9	3	7	0	5	20.0%	46.7%	Mixed
P10	0	1	2	0	0%	100.0%	L-sem
P11	31	1	1	1	91.2%	5.9%	L-phon
P12	8	3	1	5	47.1%	23.5%	Mixed
P13	4	12	5	3	16.7%	70.8%	L-sem
P14	38	2	5	1	82.6%	15.2%	L-phon
P15	6	4	1	1	50.0%	41.7%	Mixed

*Other, circumlocutions, unrelated and formal errors; %PHO, proportion of phonological errors in both paradigms; %SEM, proportion of semantic and omission errors in both paradigms; L-phon, predominant lexical-phonological error profile; L-sem, predominant lexical-semantic error profile; Mixed, mixed error profile.*

**FIGURE 1:**
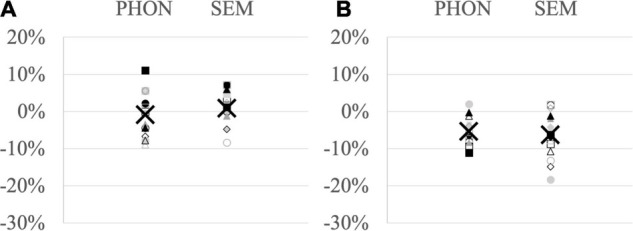
Proportion of **(A)** accuracy differences and **(B)** RT differences between related and unrelated conditions in each participant (dots, squares, diamonds, triangles) and group mean (X) for the phonological cueing paradigm (PHON – left row) and semantic cueing paradigm (SEM – right row); negative values indicate facilitation after related cues and positive values interference.

At the individual level, phonological cueing effects ranged from −8.9% facilitation to + 11.1% interference (mean −0.8%) and semantic cueing effects from −8.3% facilitation to + 7.1% interference (mean + 0.9%) (Figure 1A).

In order to run a single generalized mixed model on all data, auditory, auditory-visual and visual congruent cues were grouped as “related” trials in the phonological paradigm and associative and categorical cues were grouped as “related” trials in the semantic paradigm. The model for binomially distributed outcome was calculated with the conditions (related/unrelated trials), the paradigm (semantic/phonological cueing) and the anomic profile (lexical-semantic, lexical-phonological, mixed) as fixed factors, random intercepts by items and by participants, as well as random slopes for the condition by participants. References were the phonological paradigm, unrelated trials and the mixed anomic profile. None of the factors or interactions reached significance (all z between −1.28 and 1.30, all *ps* > 0.19) (see Supplementary Appendix III for the full model output).

### Naming Latencies

Analyses on naming latencies were performed on 3278 observations considered as correct responses. On the group average for phonological cueing, mean naming latencies were 977 ms in the control condition, 943 ms with visual cues, 912 ms with auditory-visual cues and 909 ms with auditory cues. For semantic cueing, mean naming latencies were 952 ms in the control condition, 906 ms with categorical cues and 892 ms with associative cues. Heterogeneous cueing effects were found among participants, ranging from −11.1% facilitation to + 2.0% interference (mean −5.3%) for phonological cueing and from −18.3% facilitation to + 1.9% interference for semantic cueing (mean −6.3%) (Figure 1B).

A linear regression mixed-effects model was computed on naming latencies with the same variables and the same references as for accuracy (see Supplementary Appendix III for the model syntax). Naming latencies were significantly modulated by the condition [F(1,12.15) = 69.22, *p* < 0.001] and by the anomic profile [F(2,12.02) = 5.05, *p* = 0.03], but not by the paradigm [F(1,73.42) = 3.34, *p* = 0.07]. The interaction between the condition and the anomic profile was significant [F(2,12.13) = 3.75, *p* = 0.05] but not the interaction between the condition and the paradigm [F(1,3115.64) = 1.08, *p* = 0.30], indicating that the facilitative effect of cues did not depend on the paradigm but on the anomic profile. The triple interaction between the fixed factors was significant [F(2,3113.65) = 7.06, *p* < 0.001], confirming that adding the anomic profile into the equation was critical. As for the interaction between the paradigm and the anomic profile, it was not significant [F(2,3111.82) = 1.12, *p* = 0.32]. Because of the significant triple interaction and in order to specify the impact of the different subtypes of related cues on naming latencies, separate linear mixed-effects models were calculated for each paradigm (see below).

### Naming Latencies - Phonological Cueing

**FIGURE 2:**
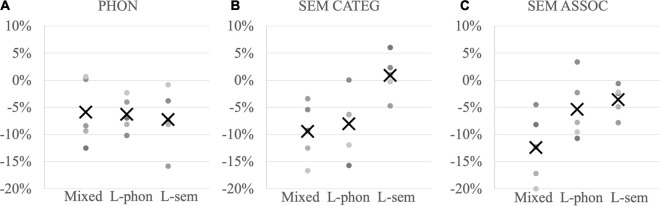
Proportion of RT differences between related and unrelated conditions in individuals (dots) per subgroup and subgroup mean (“X”) (Mixed = mixed anomic profile, L-phon = lexical-phonological anomic profile, L-sem = lexical-semantic anomic profile) **(A)** for phonological auditory and auditory-visual cueing, **(B)** categorical semantic cueing, and **(C)** associative semantic cueing; negative values indicate facilitation after related cues and positive values interference.

A linear regression mixed-effects model was computed with the four conditions (auditory cue, visual cue, auditory-visual cue, and white noise) and the three anomic profiles (lexical-semantic, lexical-phonological, mixed) as fixed factors, random intercepts for items and participants, as well as random slopes for the condition by participants. References were the control condition (white noise) and the mixed anomic profile. Main effects were significant for the condition [F(3,24.0) = 6.00, *p* = 0.003] and on the anomic profile [F(2,12.00) = 5.31, *p* = 0.02]. More precisely, pairwise comparisons showed that participants answered faster after auditory (*p* = 0.03) and auditory-visual (*p* = 0.03) phonological cues as compared to white noise, but not after cues presented only visually (*p* = 0.73). Participants with a mixed profile were overall faster than participants with lexical-semantic anomia (*p* = 0.02) or lexical-phonological anomia (*p* = 0.05). Crucially, these effects were totally independent as no interaction was found [F(6,24.00) = 0.26, *p* = 0.95], indicating that the effectiveness of phonological cues did not rely on the underlying anomic profile (Figure 2A).

### Naming Latencies - Semantic Cueing

Following the model of phonological cueing, a linear regression mixed-effects model was computed with the three conditions (associative cues, categorical cues, unrelated cues) and the three anomic profiles (lexical-semantic, lexical-phonological, mixed) as fixed factors, random intercepts for items and participants, as well as random slopes for the condition by participants. References were again the control condition (unrelated cues) and the mixed anomic profile. Main effects were significant for the condition [F(2,21.2) = 8.18, *p* = 0.002], on the anomic profile [F(2,12.0) = 3.84, *p* = 0.05] and significantly interacted [F(4,21.2) = 3.79, *p* = 0.02]. To disentangle this interaction, separate models were computed for each profile subgroup. First, participants with a mixed anomic profile (Figures 2B,C left row) showed significant semantic facilitation both after associative [t(4.21) = −3.3, SE = 0.05, *p* = 0.03; mean −13% gain on RTs] and categorical cues [t(4.20) = −3.0, SE = 0.04, *p* = 0.04; mean −10% gain on RTs]. Second, participants with lexical-phonological anomia (Figures 2B,C middle row) showed semantic facilitation after categorical cues [t(4.41) = −2.63, *p* = 0.05; mean −9% gain on RT] but not after associative cues [t(3.96) = −1.74, *p* = 0.16; mean −5% gain on RT]. Third, participants with lexical-semantic anomia (Figures 2B,C right row) did not show any semantic facilitation after associative cues [t(17.99) = −1.27, SE = 0.02, *p* = 0.22; mean −3% gain on RTs] or categorical cues [t(96.43) = 0.95, *p* = 0.35, mean + 3% loss on RTs].

In other words, auditory and auditory-visual phonological cues were effective regardless of the underlying profile (Figure 2A), semantic categorical cues for participants with a mixed profile and with lexical-phonological anomia (Figure 2B), but semantic associative cues only for participants with a mixed anomic profile (Figure 2C).

**FIGURE 3:**
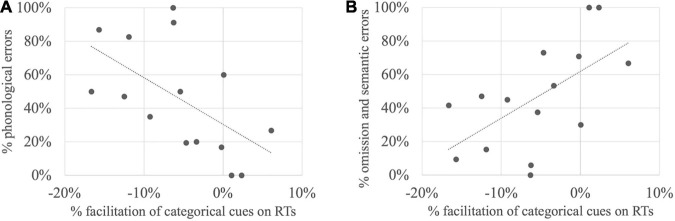
Relationship between the amount of semantic facilitation with categorical cues (each dot represents a subject around the regression line) and the amount of **(A)** phonological errors; **(B)** omission and semantic errors.

Simple linear regressions were further carried out in order to define if the amount of semantic facilitation on naming latencies by categorical or associative cues could be explained by the amount of phonological or omission/semantic errors. For categorical cues, the amount of semantic facilitation turns out to be positively related to the amount of phonological errors [F(1,13) = 6.36, *p* = 0.03, R^2^ = 0.33, R^2^_adjusted_ = 0.28] but negatively related to the amount of omission/semantic errors [F(1,13) = 7.14, *p* = 0.02, R^2^ = 0.35, R^2^_adjusted_ = 0.31]. In other terms, the more phonological the profile is, the more categorical facilitation is observed (Figure 3A), whereas the more semantic the profile is, the less categorical cues are effective (Figure 3B). In contrast for associative cues, the amount of semantic facilitation was related neither to the proportion of phonological errors [F(1,13) = 0.23, *p* = 0.64, R^2^ = 0.02, R^2^_adjusted_ = −0.05], nor to the proportion of semantic errors [F(1,13) = 0.44, *p* = 0.52, R^2^ = 0.03, R^2^_adjusted_ = −0.04].

## Discussion

In the present within-subject design study, naming accuracy and naming latencies of 15 aphasic speakers were compared after different types of phonological and semantic cues. In the phonological paradigm, auditory and auditory-visual cues corresponding to the first phoneme significantly reduced naming latencies on average across all participants. In the semantic paradigm, cues corresponding to words associatively or categorically related significantly speeded up word production only in certain cases. No effect was found on accuracy. Although the study of errors has a long tradition in aphasia research, it seems that careful measurements of naming latencies in mild anomia could also provide complementary information about the impact of cues on word retrieval following a brain lesion. The discussion will get back to the two questions raised in the Introduction.

### Comparison Between Phonological and Semantic Cueing

In group means comprising the 15 participants, there was no advantage of phonological cueing (group effect of −0.8% on errors and −5.3% on naming latencies) over semantic cueing (group effect of +0.9% on errors and −6.3% on naming latencies). Indeed, the lack of interaction between the paradigm (phonological vs. semantic) and the nature of the cues (related vs. unrelated) in statistical modeling demonstrated that semantic cues were as effective as phonological cues to facilitate word production in a heterogeneous group of aphasic speakers.

Although compatible with a previous report (Stimley and Noll, 1991), the present results diverge somewhat from other facilitation studies pointing out that phonological cueing was overall more effective than semantic cueing (Saito and Takeda, 2001; Meteyard and Bose, 2018) and several factors could explain this apparent discrepancy. First, the two latter studies did not investigate naming latencies but focused on naming accuracy, probably because anomia was more severe in their sample (no score reported in Saito and Takeda, 2001), but in Meteyard and Bose (2018) the Philadelphia Naming Test ranged from 20 to 87% vs. in the present case the adapted Boston Naming Test from 68 to 97%). In the current mild impaired aphasic speakers, comparing naming latencies turned out to be a more sensitive measure than accuracy. Second, both studies used single word cues, whereas semantic cueing was possibly maximized in the present study as two word cues were provided in half of the trials and such double priming is known to increase semantic facilitation effects (Python et al., 2018a). Third, the number of participants was slightly lower (*n* = 10 in the mentioned previous studies) as compared to the current sample size (*n* = 15) possibly reducing statistical power. Moreover, the number of trials was extremely low and unequal across the conditions in Saito and Takeda (2001), ranging from 2 to 9 trials per condition according to their experiment design where cues were provided only in case of errors and after 10 s.

### Is the Effectiveness of Different Types of Cues Related to the Underlying Anomic Profile?

In the phonological cueing paradigm, latencies were modulated by auditory and auditory-visual cues, whereas visual cues alone were insufficient to speed up picture naming. This gradient is consistent with the detailed report of Pellet Cheneval et al. (2018a). Crucially, phonological cues were efficient regardless of the anomic profile. It is worth noting that the underlying mechanisms of phonological cueing remain presently debated, as recent accounts reported that it could tap into early conceptual (e.g., Meteyard and Bose, 2018), lexical (e.g., Pellet Cheneval et al., 2018b), phonological (e.g., Roelofs, 2019) or multiple levels (e.g., Pellet Cheneval et al., 2018a) of word planning. Actually, the present data rather speaks in favor of phonological cues operating at different levels, as individuals with various anomic profiles showed indistinct phonological facilitation effects.

In the semantic cueing paradigm, different patterns of facilitation were observed on naming latencies according to the type of cue and to the anomic profile. Thereafter, results will be discussed by subgroups:

(1) In individuals with lexical-semantic anomia, both types of semantic cues (categorical and associative) were ineffective. Moreover, simple regression analyses confirmed that the more severe the lexical-semantic profile was (i.e., more omissions and semantic errors were produced), the less efficient categorical semantic cues were likely to be. It is possible that the type of semantic cues provided here (associatively or categorically related words) do not specifically operate at the semantic level albeit conceivably originating from this level. This interpretation is actually in line with electrophysiological data that we reported in young healthy participants (Python et al., 2018a): using partly the same stimuli than in the current report but presenting the cues in written form, behavioral semantic facilitation effects were linked to event-related potentials modulations in time-windows of word planning likely associated with post-lexical and monitoring processes. In terms of mechanisms at play, phonological cues strongly boosting the activation of phonological representations could bypass or retroactively trigger impaired or underspecified lexical-semantic processes. However, poor lexical-semantic processing might not be compensated by semantic cues operating post-lexically without providing any explicit phonological information about the target-word.
(2) In individuals with lexical-phonological anomia, only categorical semantic cues were significantly effective, but not associative semantic cues. Additional simple regression analyses demonstrated that the more severe the phonological profile was (i.e., more phonological errors were produced), the more efficient semantic categorical cues were likely to be. Even if categorical cues are usually inducing semantic interference if they appear almost simultaneously with the picture to name, they induce facilitation if presented sufficiently before the picture (i.e., at least −400 ms), which is the case in the current investigation. The mechanism by which categorical cues facilitate naming can be explained by semantic/conceptual priming lasting longer than activation/competition of lexical representations, that had time to decay during the “long” interval between the word and the picture (Zhang et al., 2016). The differential impact of the two types of semantic cues could lie in the amount of semantic features that they share with the target picture (Saito and Takeda, 2001). Categorical cues (e.g., lemon for the target picture “banana”) activate a number of shared semantic features (e.g., eatable, yellow, has a peel, grows on trees, …) able to strongly prime speech processing, whereas associative cues (e.g., monkey) are less prone to activate common semantic features between the cue and the target. Thanks to the quantity of shared activated features, categorical cues could boost the mapping between preserved semantics and impaired phonology more powerfully than associative cues in participants with lexical-phonological anomia.
(3) In participants with mixed lexical-semantic and -phonological anomia, both semantic associative and categorical cues were effective, as were also phonological cues. As any type of cue was speeding up picture naming, it is possible that anomia did not result from a disruption affecting a particular processing stage of word production in this subgroup. Apart of semantic and phonological disorders, a third key dimension explaining error production in aphasia seems to be related to broader executive-cognition abilities (Butler et al., 2014). Therefore, the non-specific error profile of this subgroup could either come from mild impairment in both lexical-semantic and -phonological processes, or from more global executive/monitoring limitations disturbing randomly and equally lexical-semantic and -phonological encoding. As picture naming requires both semantics (activating the concept from the picture) and phonology (encoding the word-form), it is possible that both cueing techniques could evenly increase the activation of the target by means of interactions between semantics and phonology.

### Limitations and Perspectives

Aphasia assessment before the experiment was heterogeneous, because no comprehensive aphasia battery of sufficient quality corresponding to international standards was available in French. Note that a French-speaking version of the Comprehensive Aphasia Test will hopefully be developed in the future (Fyndanis et al., 2017). Due to this lack of baseline tools, the anomic profiles were determined by the main error type during the experimental paradigms. As the presence of cues might have influenced the type of naming errors, the anomic profiles in the present study strictly reflect the most common error types produced in cued picture naming and should solely be considered as indicators of possible underlying impairments. It is thus possible that the current sample of participants could have been classified in a different way if the level of breakdown had been more clearly determined prior to testing by means of a general background assessment of language. Note also that the error rate was relatively low for some participants (e.g., P2, P7, P10) and in such mild cases, the assignment to specific anomic profiles based on a few amount of errors might be less relevant than in speakers with high error rates. As a consequence, future studies should confirm the present exploratory results in order to make robust and straightforward links between the underlying impairment and the response to phonological and semantic cues in aphasia. Finally, as phonological cueing was always presented before semantic cueing, it is not possible to exclude an order effect on the naming latencies, the type and the amount of errors.

## Conclusion

The present study revealed that phonological cues were more beneficial than semantic cues for individuals with lexical-semantic anomia, whereas both phonological and semantic cues benefited to people with a mixed anomic profile or lexical-phonological anomia. In the latter case, semantic categorical cues led to greater facilitation than semantic associative cues. Understanding the fine-grained mechanisms by which a given cue facilitates word production might upscale the theory of anomia therapy and offer new research-based therapeutic approaches in the future. Interestingly, the present data is consistent with the treatment study of van Hees et al. (2013) reporting successful phonological therapy after several weeks independently of the underlying deficit, but no benefit of semantic therapy in case of semantic impairment.

## Data Availability Statement

The raw data supporting the conclusions of this article will be made available by the authors, without undue reservation.

## Ethics Statement

The studies involving human participants were reviewed and approved by Commission cantonale (VD) d’éthique de la recherche sur l’être humain, Av. de Chailly 23, 1012 Lausanne. The participants provided their written informed consent to participate in this study.

## Author Contributions

GP, PP, and ML conceptualized the research and wrote the manuscript. GP, PP, and CB administered the tests to the participants.

## Conflict of Interest

The authors declare that the research was conducted in the absence of any commercial or financial relationships that could be construed as a potential conflict of interest.

## Publisher’s Note

All claims expressed in this article are solely those of the authors and do not necessarily represent those of their affiliated organizations, or those of the publisher, the editors and the reviewers. Any product that may be evaluated in this article, or claim that may be made by its manufacturer, is not guaranteed or endorsed by the publisher.

## Footnotes

1 Psychology Software Tools, Inc. [E-Prime 2.0]. Retrieved from http://www.pstnet.com/
