# Supplementary material for: Facilitating Word Retrieval in Aphasia: Which Type of Cues for Which Aphasic Speakers?
Source: Front Hum Neurosci. 2021 Nov 26;15:747391. doi: 10.3389/fnhum.2021.747391 (PMC8662555; doi:10.3389/fnhum.2021.747391)
Supplement: Supplementary file 1 [file Data_Sheet_1.docx]

**Supplementary Material**

**Appendix I**

**List of stimuli used in the phonological cueing paradigm (english translations provided in brackets)**

| **Target picture** | **Fillers** |
| --- | --- |
| **ballon (ball)** | balançoire (swing) |
| **bougie (candle)** | chaise (chair) |
| **bureau (desk)** | cigarette (cigarette) |
| **cerveau (brain)** | douche (shower) |
| **chaussette (sock)** | fleur (flower) |
| **chenille (caterpillar)** | jupe (skirt) |
| **cheveux (hair)** | montre (watch) |
| **citron (lemon)** | parapluie (umbrella) |
| **danseuse (dancer)** | toit (roof) |
| **dauphin (dolphin)** | verre (glass) |
| **doigt (finger)** |  |
| **fenêtre (window)** |  |
| **fourchette (fork)** |  |
| **fusée (rocket)** |  |
| **gilet (vest)** |  |
| **journal (newspaper)** |  |
| **jumelles (binoculars)** |  |
| **micro (microphone)** |  |
| **montagne (mountain)** |  |
| **moustache (mustache)** |  |
| **panier (basket)** |  |
| **pingouin (penguin)** |  |
| **poisson (fish)** |  |
| **serpent (snake)** |  |
| **tableau (board)** |  |
| **tampon (stamp)** |  |
| **taureau (taurus)** |  |
| **valise (suitcase)** |  |
| **vélo (bike)** |  |
| **voiture (car)** |  |

**Appendix II**

**List of stimuli used in the semantic cueing paradigm (english translations provided in brackets)**

| **Target picture** | **Associative cue 1** | **Associative cue 2** | **Categorical cue 1** | **Categorical cue 2** | **Unrelated cue 1** | **Unrelated cue 2** |
| --- | --- | --- | --- | --- | --- | --- |
| **avion**  **(airplane)** | vol  (flight) | air  (air) | hélicoptère (helicopter) | bus  (bus) | cordes  (ropes) | pioche  (pickaxe) |
| **balai**  **(broom)** | poussière  (dust) | nettoyage  (cleaning) | chiffon  (wipe) | aspirateur (vacuum cleaner) | acteur  (actor) | vent  (wind) |
| **banane**  **(banana)** | singe  (monkey) | peau  (skin) | citron  (lemon) | poire  (pear) | musique  (music) | éléphant  (elephant) |
| **bol**  **(bowl)** | lait  (milk) | déjeuner  (breakfast) | assiette  (plate) | plat  (dish) | jambe  (leg) | chèvre  (goat) |
| **cactus**  **(cactus)** | désert  (desert) | épine  (needle) | palmier  (palm tree) | sapin  (christmas tree) | toile  (web) | fauteuil  (armchair) |
| **caméra**  **(video camera)** | cinéma  (cinema) | vidéo  (video) | télévision (television) | micro (microphone) | intelligence  (intelligence) | tente  (tent) |
| **canon**  **(cannon)** | boulet  (cannonball) | feu  (fire) | fusil  (rifle) | pistolet  (gun) | visage  (face) | ordures  (garbage) |
| **carotte**  **(carrot)** | lapin  (rabbit) | poil  (hair) | salade  (salad) | haricot  (bean) | pique  (spine) | mouton  (sheep) |
| **clown**  **(clown)** | cirque  (circus) | rire  (laugh) | acteur  (actor) | peintre  (painter) | abeille  (bee) | bois  (wood) |
| **collier**  **(necklace)** | perle  (pearl) | diamant  (diamond) | bague  (ring) | chaîne  (chain) | point  (dot) | télévision  (television) |
| **fromage**  **(cheese)** | chèvre  (goat) | odeur  (smell) | beurre  (butter) | crème  (cream) | roi  (king) | peluche  (teddy bear) |
| **gant**  **(glove)** | main  (hand) | hiver  (winter) | veste  (jacket) | chemise  (shirt) | coq  (rooster) | apéritif  (appetizer) |
| **lion**  **(lion)** | roi  (king) | crinière  (mane) | tigre  (tiger) | chat  (cat) | papier  (paper) | nouvelles  (news) |
| **lit**  **(bed)** | endormissement  (falling sleep) | sommeil  (sleep) | fauteuil (armchair) | table  (table) | neige  (snow) | crayon  (pencil) |
| **maison**  **(house)** | famille  (family) | toit  (roof) | immeuble  (building) | appartement  (flat) | oeuf  (egg) | palmier  (palm tree) |
| **marteau**  **(hammer)** | clou  (nail) | tape  (hit) | tournevis  (screwdriver) | pince  (pliers) | souris  (mouse) | vol  (flight) |
| **peigne**  **(comb)** | cheveux  (hair) | coiffure  (haircut) | brosse  (brush) | savon  (soap) | mare  (pond) | citron  (lemon) |
| **pied**  **(foot)** | chaussure  (shoe) | marche  (walk) | jambe  (leg) | bras  (arm) | scooter  (scooter) | lait  (milk) |
| **poubelle**  **(dustbin)** | ordures  (garbage) | déchets  (waste) | carton  (carton) | bac  (tray) | friture  (frying) | loup  (wolf) |
| **stylo**  **(pen)** | bille  (ball) | encre  (ink) | feuille  (sheet) | livre  (book) | requin  (shark) | bruit  (noise) |
| **violon**  **(violin)** | musique  (music) | archet  (bow) | guitare  (guitar) | flûte  (flute) | camion  (truck) | jardin  (garden) |

**Appendix III**

**Full models (syntax and outputs)**

**Generalized mixed model on accuracy**

*Input:*

glmer(accuracy ~ condition * paradigm * profile + (1|item) + (1|participant) + (1+condition|participant), data = data15participants, family=binomial, control=glmerControl(optimizer="bobyqa"))

*Output :*

Fixed effects:

Estimate Std. Error z p

condition unrelated vs related -0.373020 0.291025 -1.282 0.200

paradigm semantic vs phonological 0.271312 0.277003 0.979 0.327

profile mixed vs lexical-phonemic -0.287180 0.576331 -0.498 0.618

profile mixed vs lexical-semantic -0.003138 0.584391 -0.005 0.996

condition*paradigm 0.435423 0.427995 1.017 0.309

condition*profile mixed vs lexical-phonemic 0.281989 0.401686 0.702 0.483

condition*profile mixed vs lexical-semantic 0.568867 0.436768 1.302 0.193

paradigm*profile mixed vs lexical-phonemic -0.134932 0.315605 -0.428 0.669

paradigm*profile mixed vs lexical-semantic 0.078608 0.328932 0.239 0.811

condition*paradigm*profile mixed vs lexical-phonemic -0.446457 0.569558 -0.784 0.433

condition*paradigm*profile mixed vs lexical-semantic -0.168952 0.624341 -0.271 0.787

**Mixed-effects model on naming latencies**

*Input:*

lmer(log(RT) ~ condition * paradigm * profile + (1|item) + (1|participant) + (1+condition|participant), data = data15participants)

*Output :*

Main effects (Type III Analysis of Variance Table with Satterthwaite's method)

Sum Sq Mean Sq NumDF DenDF F p

condition unrelated vs related 2.47908 2.47908 1 12.15 69.2228 2.306e-06

paradigm semantic vs phonological 0.11971 0.11971 1 73.42 3.3425 0.0715736

profile (3 levels) 0.36198 0.18099 2 12.02 5.0537 0.0255450

condition*paradigm 0.03879 0.03879 1 3115.64 1.0832 0.2980700

condition*profile 0.26868 0.13434 2 12.13 3.7511 0.0539339

paradigm*profile 0.08054 0.04027 2 3111.82 1.1244 0.3249713

condition*paradigm*profile 0.50592 0.25296 2 3113.65 7.0634 0.0008697

**Mixed-effects model on naming latencies for the phonological paradigm**

*Input:*

lmer(log(RT) ~ condition * profile + (1|item) + (1|participant) + (1+condition|participant), data = data15participants_phonological)

*Output :*

Main effects (Type III Analysis of Variance Table with Satterthwaite's method)

Sum Sq Mean Sq NumDF DenDF F p

condition (4 levels) 0.73956 0.246519 3 24.015 5.9988 0.003355

profile (3 levels) 0.43637 0.218186 2 11.997 5.3093 0.022304

condition*profile 0.06356 0.010594 6 24.004 0.2578 0.951131

Fixed effects:

Estimate Std. Error df t p

condition auditory vs control -0.088790 0.035372 13.286634 -2.510 0.0257

condition auditory-visual vs control -0.076687 0.032426 16.116564 -2.365 0.0309

condition visual vs control -0.009500 0.026972 44.526829 -0.352 0.7263

profile mixed vs lexical-phonemic 0.218536 0.097887 12.013358 2.233 0.0454

profile mixed vs lexical-semantic 0.273800 0.097829 11.984411 2.799 0.0161

condition auditory vs control*profile mixed vs lex-phon 0.007591 0.050383 13.646422 0.151 0.8824

condition auditory-visual vs control*profile mixed vs lex-phon 0.008980 0.045900 16.186179 0.196 0.8473

condition visual vs control*profile mixed vs lex-phon -0.007745 0.038232 44.936727 -0.203 0.8404

condition auditory vs control*profile mixed vs lex-sem 0.016863 0.050011 13.253526 0.337 0.7413

condition auditory-visual vs control*profile mixed vs lex-sem 0.003049 0.045887 16.136287 0.066 0.9478

condition visual vs control*profile mixed vs lex-sem -0.036853 0.038070 44.193261 -0.968 0.3383

**Mixed-effects model on naming latencies for the semantic paradigm (15 participants)**

*Input:*

lmer(log(RT) ~ condition * profile + (1 |item) + (1 |participant) + (1+condition|participant), data = data15participants_semantic)

*Output :*

Main effects (Type III Analysis of Variance Table with Satterthwaite's method)

Sum Sq Mean Sq NumDF DenDF F p

condition (3 levels) 0.92896 0.46448 2 21.167 8.1788 0.002335

profile (3 levels) 0.43615 0.21808 2 12.006 3.8400 0.051381

condition*profile 0.86159 0.21540 4 21.162 3.7928 0.017783

**Mixed-effects model on naming latencies for the semantic paradigm (5 participants with mixed profile)**

*Input:*

lmer(log(RT) ~ condition + (1|item) + (1|participant) + (1+condition|participant), data = data5participantsMIXED_semantic)

*Output :*

Fixed effects:

Estimate Std. Error df t p

condition associative vs unrelated -0.16938 0.05057 4.20942 -3.349 0.0265

condition categorical vs unrelated -0.12886 0.04229 4.19770 -3.047 0.0358

**Mixed-effects model on naming latencies for the semantic paradigm (5 participants with lexical-phonemic profile)**

*Input:*

lmer(log(RT) ~ condition + (1|item) + (1|participant) + (1+condition|participant), data = data5participantsPHO_semantic)

*Output :*

Fixed effects:

Estimate Std. Error df t p

condition associative vs unrelated -0.08122 0.04664 3.96242 -1.742 0.1572

condition categorical vs unrelated 0.10981 0.04171 4.40923 -2.633 0.0526

**Mixed-effects model on naming latencies for the semantic paradigm (5 participants with lexical-semantic profile)**

*Input:*

lmer(log(RT) ~ condition + (1|item) + (1|participant) + (1+condition|participant), data = data5participantsSEM_semantic)

*Output :*

Fixed effects:

Estimate Std. Error df t p

condition associative vs unrelated -0.02870 0.02259 17.99715 -1.270 0.220

condition categorical vs unrelated 0.02015 0.02132 96.36779 0.945 0.347
